# Renal Embolism Associated with the Atrial Myxoma: A Case Report and Literature Review

**DOI:** 10.3390/medicina60050694

**Published:** 2024-04-24

**Authors:** Masatoshi Sega, Marina Yamashita, Hiroshi Maruyama, Yuji Taya, Kentaro Ohgi, Rei Haraoka, Kouichi Hirayama

**Affiliations:** 1 Department of Nephrology, Tokyo Medical University Ibaraki Medical Center, Ami 300-0395, Ibaraki, Japan; 2 Department of Cardiology, Tokyo Medical University Ibaraki Medical Center, Ami 300-0395, Ibaraki, Japan; 3 Department of Intensive Care Medicine, Tokyo Medical University Ibaraki Medical Center, Ami 300-0395, Ibaraki, Japan; 4 Department of Neurosurgery, Tokyo Medical University Ibaraki Medical Center, Ami 300-0395, Ibaraki, Japan

**Keywords:** renal infarction, renal embolism, cardiac myxoma, hemodialysis

## Abstract

Renal embolisms due to cardiac myxomas are extremely rare; the clinical course, treatment, and prognosis of this disease are not established. A 69-year-old Japanese woman who underwent a nephrectomy for renal cell carcinoma 3 years earlier was hospitalized with a right occipital lobe cerebral infarction. Her renal function suddenly worsened 3 days post-admission: her serum creatinine rose from 1.46 mg/dL to 6.57 mg/dL and then to 8.03 mg/dL the next day, and hemodialysis therapy was started. Abdominal computed tomography (CT) scans showed patchy non-contrasted low-density areas in the right kidney, and chest CT scans and transesophageal ultrasonography revealed a left atrial tumor. We diagnosed renal infarction due to a left atrial myxoma. Hemodialysis and anticoagulant therapy (heparin) were continued, followed by the cardiac myxoma’s resection. The patient’s renal function gradually improved post-surgery, and the hemodialysis was discontinued. Considering our patient and 19 other case reports of renal infarction associated with cardiac myxoma, the treatment for such a renal infarction and the outcomes differ depending on the embolus site. The poor outcome of abdominal aortic embolism requires a prompt embolectomy, whereas a branch renal artery embolism requires anticoagulation therapy to prevent thrombosis formation around the myxoma.

## 1. Introduction

A renal infarction is caused by a unilateral or bilateral occlusion of the main trunk of a renal artery or its branches that is due to an embolus or thrombus, resulting to peripheral circulation disorders and necrosis of renal tissue. The exact prevalence of renal infarction is unclear, but a recent 7-year epidemiological study showed an increasing trend from 2.68 to 3.06 per 100,000 person–years [1]. Renal infarctions are classified into four groups depending on their cause: cardiogenic (embolic), renal artery injury (renovascular), hypercoagulability disorders, and idiopathic infarctions [2]. The frequency of renal infarction by cause varies depending on the report, and the frequency of cardiogenic renal infarction is reported to be approx. 10–50% [1,2,3,4,5].

The most common etiology of cardioembolic diseases (including ischemic stroke) is atrial fibrillation with or without valvular heart disease [6,7]. In addition to atrial fibrillation and valvular heart disease, infective endocarditis is also well known as a cause of cardiogenic embolism, but cardiac tumors are a rare cause.

Primary cardiac tumors are rare, with reported incidence rates of 0.0017–0.33% in autopsy cases [8] and that of 1.38 in 100,000 individuals per year from an Italian multicenter cohort study [9]. A myxoma is the most frequent type of primary cardiac tumor, accounting for approx. 70% of them [10]. However, cardiac myxoma is rare; the prevalence of cardiac myxoma is approximately 0.03% in the general population [11], and incidence of cardiac myxoma is 0.05 to 0.16 of 100,000 individuals per year [12,13]. In Japan, there has been no change in the incidence rate of cardiac myxoma over the past 20 years [14]. A cohort study of 112 cases of left atrial myxoma indicated that 30% of the cases included an embolism, but the majority were cerebrovascular disorders, and there were no cases of renal artery embolism [15]. Another cohort study of 207 cardiac myxoma cases revealed that the incidence of embolism was 15.5% (32/207), with the majority being cerebral artery embolisms (54%, 20/37) and renal embolism being extremely rare, with only a single case [16].

We experienced a rare case in which a patient who had only a single kidney due to nephrectomy for renal cell carcinoma developed renal infarction due to myxoma. In this patient’s case, hemodialysis therapy was temporarily required because the patient had a solitary kidney, but her renal function improved with anticoagulant therapy and tumor removal, and the hemodialysis therapy was then withdrawn. A renal embolism due to a cardiac myxoma is extremely rare. We provide the case details and the clinical course of this patient, and we review the 19 reported cases of renal infarction associated with cardiac myxoma.

## 2. Case Presentation

A 69-year-old Japanese woman came to our hospital on 25 December 2021 because of headache and nausea. Twenty years earlier she had been diagnosed with hypertension and diabetes and had been under treatment with telmisartan, amlodipine, vildagliptin, and metformin since then. Three years before her present admission, she underwent a left nephrectomy for a left renal cell carcinoma. None of her family members had a history of heart or renal disease, but her father had a history of gastric cancer. The patient had no tobacco smoking or alcohol consumption habit.

**Figure 1:**
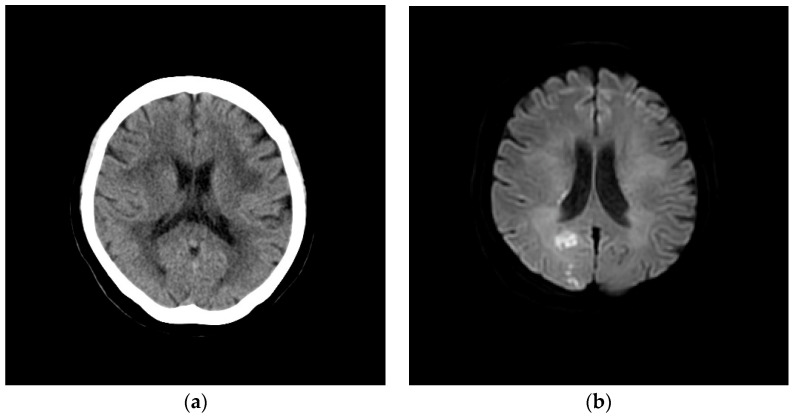
The brain computed tomography (**a**) and magnetic resonance imaging with diffusion-weighted imaging (**b**) results of the patient, a 69-year-old woman.

The physical examination confirmed the patient’s fever and revealed anemic conjunctiva; her blood pressure was 167/109 mmHg, and her pulse rate was 78 bpm with regular rhythm. The oxygen saturation was 96% via pulse oximetry on room air. In laboratory examinations, the blood urea nitrogen and serum creatinine levels were elevated at 23.9 and 1.46 mg/dL, respectively. The serum levels of lactate dehydrogenase (LDH) (172 U/mL) and C-reactive protein (CRP) (0.23 mg/dL) were within normal limits. Electrocardiography revealed a regular sinus rhythm with no abnormal findings. A chest radiograph showed no apparent pulmonary parenchymal abnormality and no cardiomegaly. A brain computed tomography (CT) examination showed no abnormal-density areas (Figure 1a), but magnetic resonance imaging (MRI) with diffusion-weighted imaging detected several high-intensity areas in the right occipital lobe (Figure 1b).

**Figure 2:**
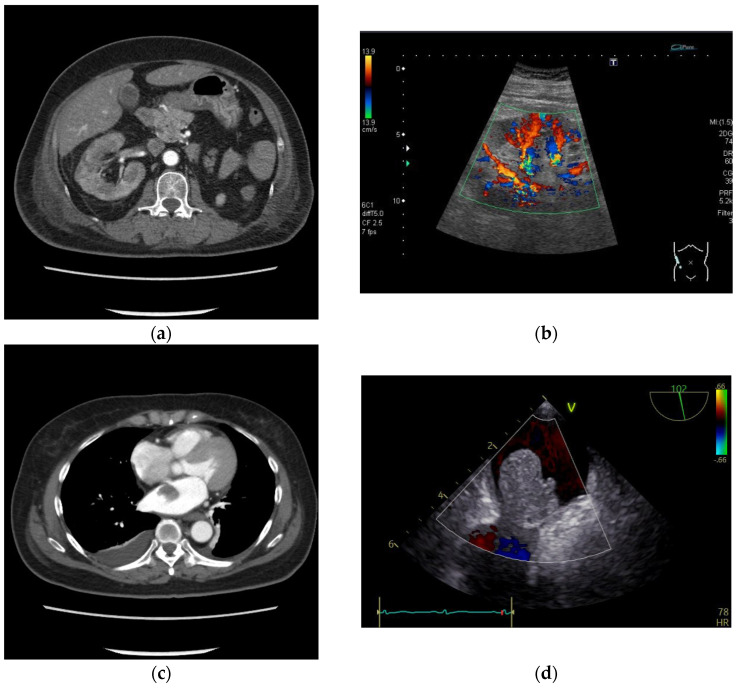
The patient’s enhanced abdominal computed tomography scan (**a**), renal ultrasonography (**b**), enhanced chest computed tomography scan (**c**), and transesophageal ultrasonography (**d**).

Three days after the patient’s admission, oliguria was observed, and her renal function rapidly worsened; the blood urea nitrogen and serum creatinine (Cr) levels were elevated at 68.1 and 6.57 mg/dL, respectively. Leukocytosis, mild anemia, and elevated levels of LDH (1901 U/mL) and CRP (26.6 mg/dL) were also observed (Table 1). The oliguria did not improve even with fluid replacement, and the serum Cr level further increased to 8.03 mg/dL the next day; we thus initiated hemodialysis with a catheter inserted into the patient’s internal jugular vein. After the initiation of hemodialysis, a contrast medium-enhanced abdominal CT examination showed non-contrasted low-density areas in the right kidney (Figure 2a), but renal ultrasonography showed partial preservation of renal blood flow (Figure 2b). A cardiac tumor attached to the left atrial septum was observed in both the enhanced chest CT scan (Figure 2c) and transesophageal ultrasonography (Figure 2d). Based on the above findings, we diagnosed a renal infarction due to a left atrial myxoma.

Hemodialysis using heparin (4000 U/session) as anticoagulant therapy was continued 3×/week, and heparin was also administered to prevent a further embolism (5000–10,000 U/day). At 18 days after the patient’s admission, a resection of the tumor with the surrounding atrial septum and reconstruction of the defects using an expanded polytetrafluoroethylene (ePTFE) patch were performed. The histopathological examination of the tumor revealed diffuse, honeycomb, cord, and luminal proliferations of spindle- and star-shaped cells in a mucinous or fibrinous tissue.

The patient’s postoperative course progressed with no complications, and her urine output gradually increased after the surgery. Her serum Cr level gradually decreased, and the hemodialysis therapy was withdrawn on the 20th postoperative day. Approximately 2 years have passed since the onset of the disease, but the patient’s serum Cr level has remained at ~3 mg/dL.

## 3. Discussion

The majority of cardiac myxomas occur sporadically, while 30–50% of cases develop as a part of Carney complex syndrome [17]. Carney complex is characterized by skin pigmentary abnormalities, myxomas, endocrine tumors or overactivity, and schwannomas [18]. Carney complex is an inheritable and autosomal dominant condition, and germline mutations in the gene coding the protein kinase A regulatory subunit 1 alpha (PRKAR1A) located on the locus 17q22-24 were responsible for several phenotypes of this disease [19]. The present case differs from Carney complex as it involved an advanced age, had no genetic history, and did not have lesions characteristic of this syndrome other than cardiac myxoma, such as skin lesions, endocrine abnormalities, or neurological lesions. It has been reported that genetic abnormalities may be observed even in sporadic cases [20]. However, genetic examination was not performed in the present case, so the involvement of genetic abnormalities of PRKAR1A is unknown.

In the present case, histological diagnosis has been made for cardiac tumors, but not for cerebral infarction and renal infarction. This case is relatively elderly and has a history of diabetes and hypertension, so the possibility of cerebral infarction and renal infarction due to arteriosclerosis cannot be ruled out. However, infarctions in the brain and kidneys were observed at the same time despite no dehydration, and imaging tests showed infarctions in multiple areas of the brain and in a branch of renal artery, not in the main trunk, leading to a diagnosis of renal artery embolism due to myxoma. Additionally, the fact that renal infarction occurred despite the patient taking anticoagulant therapy after the cerebral infarction may support the possibility of embolism caused by cardiac myxoma.

Cases of renal infarction due to a left atrial myxoma are rare. We searched the available literature up to 31 December 2023 in the PubMed^®^ from MEDLINE electronic databases, using the key words ‘cardiac myxoma’ and ‘renal artery infarction’ or ‘renal artery embolism’ (Supplementary File Table S1). We detected 42 reports, and 10 were excluded as they were in a language other than English. Among the remaining 32 reports, four were excluded because the subjects did not have a myxoma, and eight case reports of patients without a renal infarction/embolism were excluded. The first reported case of a renal embolism due to a myxoma was described in 1947 [21], but it was not possible to obtain the manuscript itself due to its date. A final total of 20 case reports (Supplementary Table S2) [22,23,24,25,26,27,28,29,30,31,32,33,34,35,36,37,38,39,40] including our patient’s case were thus obtained and analyzed (Supplementary File Figure S1).

The patients with a renal infarction/embolism due to a cardiac myxoma were 9 males and 11 females, with the mean age 44.4 years (range 21–70 years). Back or flank pain is a common initial symptom of renal infarction, and although such pain was observed in 20% of the 19 patients, the most common symptoms were stroke symptoms such as paralysis (60%) and lower-limb ischemic symptoms such as lower-limb pain and dysesthesia (55%). Those initial symptoms were due to complications of the embolism other than the renal embolism, with a cerebral artery embolism occurring in 60% (12/20), splenic artery emboli in 40% (8/20), and lower-extremity artery emboli in 55% (11/20) of the patients.

The cardiac myxoma originated from the left atrium in the majority of the cases (19/20), and the remaining patient’s cardiac myxoma originated from the both the right and left atrium. The size of the myxoma remaining in the atrium varied and it does not correlate with the patient’s clinical symptoms, suggesting that other factors contributed to disease severity.

The renal infarction was on the right side in 17% (3/18), on the left side in 28% (5/18), and on both sides in 56% (10/18) of the cases. The embolization site was the abdominal aorta in 17% (3/18), the renal artery main trunk in 28% (5/18), and the renal artery branch in 56% (10/18) of the cases. The serum Cr value was described in only 6 patients, and thus details regarding the other patients’ renal function were unknown. Of the patients, 5 required dialysis therapy, and 3 of these patients had abdominal aortic involvement. In one of the remaining 2 cases, renal-artery thrombosis occurred on the opposite side of an atrophic kidney, and the other case was our patient, in whom an infarction in a branch of the renal artery occurred on the opposite side after a nephrectomy. We speculate that for our patient, the reason why dialysis treatment was required despite the presence of an infarcted branch is because an embolus was first formed in the main renal artery of the residual kidney, leading to decreased renal function. We suspect that the embolus subsequently decomposed due to its natural course or due to anticoagulant therapy, causing a renal infarction of multiple branches. Acute kidney injury had occurred due to renal ischemia, and this may be why the patient’s renal function did not improve immediately.

Based on the possibility that the prognosis for these 20 patients differed depending on the location of the renal embolism, we classified the patients into three groups according to the location of the embolus (Table 2). Our evaluation of the cases revealed no significant differences among the three groups regarding gender or age, but the rate of requiring dialysis therapy was significantly higher in the patients who had undergone abdominal aortic embolization, which was performed in all of the patients in that embolus-location group. In the patients who had undergone abdominal aortic embolization, ischemia of the lower-extremity arteries was present in all cases and superior mesenteric artery lesions in 67%, thus requiring a prompt embolectomy in all cases. In contrast, among the patients with a renal artery branch infarction, embolectomy was performed only for lesions other than the renal artery in a small number of cases, and anticoagulation therapy was frequently performed.

Regarding the patients’ prognoses, death was reported in one patient, who had undergone abdominal aortic embolization due to intestinal ischemia and one patient with a main renal artery infarction due to a severely anoxic brain. The renal prognosis of the complete patient series was relatively good, and although there was one case in which a nephrectomy was performed due to stenosis of the main renal artery, there were no cases in which maintenance dialysis therapy was required. Of the five patients who required temporary hemodialysis, the hemodialysis treatment was discontinued for all except the patient who died.

## 4. Conclusions

Considering the present patient and other case reports of a renal infarction associated with a cardiac myxoma, the treatment for a renal infarction associated with a cardiac myxoma differs depending on the site of the embolus. An abdominal aortic embolism requires a prompt embolectomy, whereas a branch renal artery embolism requires anticoagulation therapy to prevent the formation of a thrombosis around the myxoma.

## Figures and Tables

**Table 1:** Laboratory data before the initiation of hemodialysis.

**Urinalysis**			**Metabolic endocrinology**		
	Protein (g/gCr)	1.02		LDL-cholesterol (mg/dL)	80
	Occult blood	2+		HDL-cholesterol (mg/dL)	49
	Red blood cell (/hpf)	1–4		Triglyceride (mg/dL)	203
	N-acetyl glucosamine (U/L)	10.2		Blood sugar (mg/dL)	140
	β2-microglobulin (μg/L)	97,900		Hemoglobin A1c (%)	6.8
**Blood cell count**				Free triiodothyronine (pg/mL)	1.45
	White blood cell (/μL)	12,100		Free thyroxine (ng/dL)	0.99
	Hemoglobin (g/dL)	10.3		Thyroid stimulating hormone (μU/mL)	0.35
	Platelet (×10^4^/μL)	17.1	**Serology**		
**Coagulation**				C-reactive protein (mg/dL)	26.6
	Prothrombin time (INR)	1.21		Immunoglobulin G (mg/dL)	939.1
	APTT (sec)	30.1		Immunoglobulin A (mg/dL)	146.5
	Fibrinogen (mg/dL)	650		Immunoglobulin M (mg/dL)	30.9
	Fibrin degradation products (μg/mL)	4.0		Immunoglobulin E (U/mL)	412
**Chemistry**				Complement factor 3 (mg/dL)	135.5
	Total protein (g/dL)	6.1		Complement factor 4 (mg/dL)	28.6
	Albumin (g/dL)	3.1		CH50 (U/mL)	38.3
	Aspartate aminotransferase (U/L)	63		Anti-streptolysin O antibody (U/mL)	12.3
	Alanine aminotransferase (U/L)	60		Rheumatoid factor (U/mL)	7.9
	Lactate dehydrogenase (U/L)	1901		Antinuclear antibody	negative
	Creatine kinase (U/L)	120		MPO-ANCA (U/mL)	<1.0
	Blood urea nitrogen (mg/dL)	68.1		PR3-ANCA (U/mL)	<1.0
	Creatinine (mg/dL)	6.85		Anti-GBM antibody (U/mL)	<2.0
	Uric acid (mg/dL)	9.2		Hepatitis B virus surface antigen	negative
	Sodium (mEq/L)	138		Anti-hepatitis C virus antibody	negative
	Potassium (mEq/L)	4.5		Rapid plasma reagin	negative
	Chloride (mEq/L)	106		Treponema pallidum hemagglutination	negative

APTT, activated partial thromboplastin time; CH50, 50% hemolytic unit of complement; MPO, myeloperoxidase; PR3, proteinase-3; ANCA, antineutrophil cytoplasmic antibody; GBM, glomerular basement membrane.

**Table 2:** Characteristics, treatment, and prognosis of renal infarction cases associated with cardiac myxoma.

	Abdominal Aorta n = 3	Main Renal Artery n = 5	Branch of Renal Artery n = 10
Age	35.3 ± 18.3 (21–56)	39.2 ± 19.7 (21–70)	50.9 ± 13.7 (24–69)
Male gender	67% (2/3)	40% (2/5)	40% (4/10)
Laterality of renal artery:			
Right	0% (0/3)	20% (1/5)	20% (2/10)
Left	33% (1/3)	40% (2/5)	20% (2/10)
Bilateral	67% (2/3)	40% (2/5)	60% (6/10)
Other involved arteries:			
Cerebral	33% (1/3)	60% (3/5)	60% (6/10)
Coronary	0% (0/3)	40% (2/5)	10% (1/10)
Splenic	67% (2/3)	20% (1/5)	40% (4/10)
Superior mesenteric	67% (2/3)	20% (1/5)	0% (0/10)
Lower limb	100% (3/3)	60% (3/5)	40% (4/10)
Serum creatinine, mg/dL			
At onset	1.99 (n = 1)	1.23 (n = 2)	1.33 (n = 3)
Maximum	2.16 (n = 1)	3.04 (n = 1)	8.42 (n = 2)
Dialysis	100% (3/3)	20% (1/5)	10% (1/10)
Treatment:			
Embolectomy	100% (3/3)	60% (3/5)	30% (3/10)
Embolectomy for renal artery	100% (3/3)	40% (2/5)	0% (0/10)
Thrombolysis	33% (1/3)	60% (3/5)	0% (0/10)
Anticoagulant	33% (1/3)	40% (2/5)	70% (7/10)
Outcome:			
Survival rate	67% (2/3)	80% (4/5)	100% (10/10)
Renal survival rate	67% (2/3)	60% (3/5)	100% (10/10)

## Data Availability

Datasets examined in this report are available from the corresponding author on reasonable request.

## Supplementary Materials

The following supporting information can be downloaded at: https://www.mdpi.com/article/10.3390/medicina60050694/s1, Table S1: Details of the search strategy used in this literature review; Table S2: Characteristics of the present and reported cases of patients with a renal infarction associated with a cardiac myxoma; Figure S1: The flowchart of the screening process in this literature review.
