# Supplementary material for: Renal Embolism Associated with the Atrial Myxoma: A Case Report and Literature Review
Source: Medicina (Kaunas). 2024 Apr 24;60(5):694. doi: 10.3390/medicina60050694 (PMC11123329; doi:10.3390/medicina60050694)
Supplement: Supplementary file 1 [file medicina-60-00694-s001.zip › Table S1.pdf]

Table S1. Details of the search formula used in this review.

No.	the search formula
1	(("heart"[MeSH Terms] OR "heart"[All Fields] OR "cardiac"[All Fields]) AND ("myxoma"[MeSH Terms] OR "myxoma"[All Fields])) AND (("renal artery"[MeSH Terms] OR ("renal"[All Fields] AND "artery"[All Fields]) OR "renal artery"[All Fields]) AND ("infarction"[MeSH Terms] OR "infarction"[All Fields]))
2	(("heart"[MeSH Terms] OR "heart"[All Fields] OR "cardiac"[All Fields]) AND ("myxoma"[MeSH Terms] OR "myxoma"[All Fields])) AND (("renal artery"[MeSH Terms] OR ("renal"[All Fields] AND "artery"[All Fields]) OR "renal artery"[All Fields]) AND ("embolism"[MeSH Terms] OR "embolism"[All Fields]))
