# Supplementary material for: Renal Embolism Associated with the Atrial Myxoma: A Case Report and Literature Review
Source: Medicina (Kaunas). 2024 Apr 24;60(5):694. doi: 10.3390/medicina60050694 (PMC11123329; doi:10.3390/medicina60050694)
Supplement: Supplementary file 1 [file medicina-60-00694-s001.zip › Table S2.pdf]

Table S2: Characteristics of the present and reported cases of patients with a renal infarction associated with a cardiac myxoma.

Ref. No.	Authors	Year	Age	Gender	Initial symptom	Myxoma	
						Site	Size, mm
22	Mattle HP et al.	1995	49	M	hemiplegia aphasia	left artium	NA
23	Loche F et al.	1999	53	F	hemiplegia	left artium	NA
24	Fujisaki K et al.	2007	24	M	right flank pain	left artium	39 x 49
25	Blackmon SH et al.	2010	23	M	right flank pain	left artium	100 x 90 x 40
26	Vogel B et al.	2011	43	F	hemiparesis<br>limb pain	left artium	10 x 5 x 5
27	Nicholls GM et al.	2012	44	M	back pain<br>altered mental state	left artium	55 x 45 x 35
28	Kim H et al.	2012	58	F	right flank pain	left artium	NA
29	Zhang T et al.	2012	29	M	limb pain<br>paresthesia	left artium	3
30	Huang CY et al.	2012	56	M	paraplegia<br>hypoesthesia	left artium	75 x 30 x 15
31	Mackie BD et al.	2014	39	F	unresponsive respiratory distress	left artium	20 x 25
32	Min SY et al.	2014	36	F	limb pain altered mental status	bilateral atrium	40 x 40 x 30<br>50 x 40 x 30
33	Michaud M et al.	2015	51	F	dysesthesia	left artium	50 x 48
34	McGowan AR et al.	2016	21	F	seizure	left artium	NA
35	Ji L et al.	2017	68	M	shortness of breath	left artium	NA
36	Silva MA et al.	2018	21	F	stroke	left artium	NA
37	Ho KKF et al.	2020	50	F	right leg pain paresthesia	left artium	disappeared
38	Shrestha S et al.	2020	55	M	dizziness headache vomiting gait disturbance	left artium	34 x 32
39	Ranjan P et al.	2021	54	F	fever vomiting abdominal pain	left artium	41 x 21
40	Han S et al.	2023	70	M	left flank pain	left artium	26 x 24 x 22

M, male; F, female; NA, not available

Ref. No.	Renal infarction		Serum Cr, mg/dL		Dialysis	Other involved arteries	Previous disease
	Vascular size	Laterality	At onset	Max.			
22	NA	NA	NA	NA	no	cerebral, lower limbs	no
23	NA	NA	NA	NA	no	cerebral, splenic, cutaneous	no
24	branch of renal artery	right	0.74	NA	no	no	no
25	main renal artery	right	NA	NA	no	no	no
26	main renal artery	bilateral	NA	NA	no	cerebral, coronary, abdominal aorta, splenic, lower limbs	no
27	branch of renal artery	bilateral	NA	NA	no	carotid, abdominal aorta, splenic, lower limbs	no
28	branch of renal artery	bilateral	NA	NA	no	cerebral, coronary anurysms	stroke
29	abdominal aorta	bilateral	NA	NA	yes	abdominal aorta, splenic, lower limbs	no
30	abdominal aorta	bilateral	1.99	2.16	yes	abdominal aorta	no
31	main renal artery	bilateral	NA	NA	no	cerebral, coronary, common iliac	no
32	branch of renal artery	bilateral	NA	NA	no	cerebral, splenic, common iliac, lower limbs	no
33	branch of renal artery	bilateral	NA	NA	no	cerebral, splenic, upper and lower limbs	HT, osteopenia
34	abdominal aorta main renal artery	left	NA	NA	yes	cerebral, abdominal aorta, SMA, lower limbs	no
35	branch of renal artery	left	1.79	8.81	no	no	IHD, CHF, Af, Rt renal atrophy
36	main renal artery	left	1.06	3.04	yes	cerebral, SMA, lower limbs	no
37	branch of renal artery	bilateral	NA	NA	no	bilateral lower limbs	no
38	branch of renal artery	bilateral	NA	NA	no	cerebral, splenic	no
39	branch of renal artery	left	NA	NA	no	no	HT, DM, IHD, IDA, hypothyroid
40	main renal artery	left	1.4	NA	no	no	HT, HL, CHF, IHD, kidney stone

Cr, creatinine; NA, not available; SMA, superior mesentric artery; HT, hypertension; IHD, ischemic heart disease; CHF, congestive heart failure; Af, atrial fibrilation; DM, diabetes melitus; IDA, iron defeciency anemia; HL, hyperlipidemia.

Ref. No.	Treatments			Outcome	
	Embolectomy	Thrombolysis	Anticoagulant	Survival	Renal survival
22	no	no	no	alive	-
23	no	no	no	alive	-
24	no	no	heparin	alive	-
25	no	no	no	alive	nephrectomy
26	yes (aorta, Rt renal, iliac)	urokinase	heparin	alive	-
27	yes (iliac)	no	heparin	alive	-
28	no	no	no	alive	-
29	yes (femoral)	urokinase	no	alive	improved
30	yes (aorta)	no	no	died	died
31	yes (coronary)	tPA	NA	died	died
32	yes (common iliac, limb)	NA	NA	alive	improved
33	no	no	no	alive	-
34	yes (MCA, aorta, femoral)	no	heparin	alive	improved
35	no	no	heparin + warfarin	alive	improved
36	yes (MCA, renal, femoral, etc.)	tPA	NA	alive	improved
37	yes (popliteal)	no	heparin	alive	-
38	no	no	heparin	alive	-
39	no	no	heparin	alive	-
40	no	no	heparin	alive	improved

NA, not available; MCA, middle cerebral artery; tPA, tissue plasminogen activator
