# Supplementary figures and images for: Renal Embolism Associated with the Atrial Myxoma: A Case Report and Literature Review
Source: Medicina (Kaunas). 2024 Apr 24;60(5):694. doi: 10.3390/medicina60050694 (PMC11123329; doi:10.3390/medicina60050694)

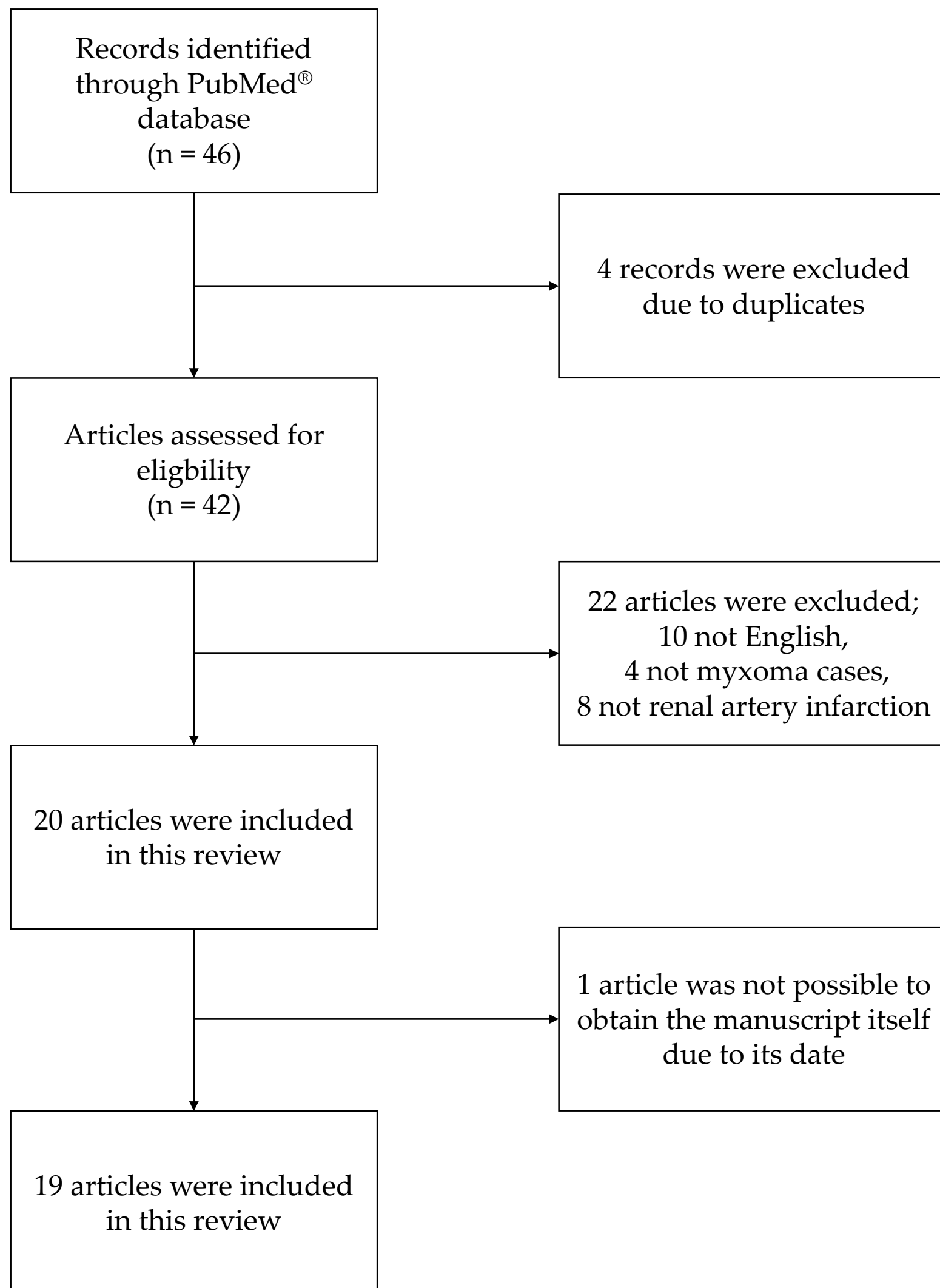

**Figure S1: The flowchart of the screening process in this literature review.**

Supplement: Supplementary file 1 [file medicina-60-00694-s001.zip › Figure S1.pdf]
